# Kimura disease in children: A report of 11 cases and review of the literature

**DOI:** 10.3389/fped.2023.1131963

**Published:** 2023-02-17

**Authors:** Yumiao Mai, Yingjie Wang, Pan Sun, Zhaohe Jing, Pengpeng Dong, Jian Liu

**Affiliations:** Department of Pediatrics, The First Affiliated Hospital of Zhengzhou University, Zhengzhou, China

**Keywords:** Kimura disease, eosinophilic hyperplastic lymphoid granuloma, eosinophilia, treatment, children

## Abstract

**Introduction:**

Kimura disease (KD), also known as eosinophilic hyperplastic lymphoid granuloma, is a rare benign chronic inflammatory condition, which is featured with the painless progressive mass located in the subcutaneous area of the head and neck region, elevated peripheral blood eosinophils, and raised serum immunoglobulin E (IgE) levels. KD is uncommon in clinical practice, especially with low incidence in children, so it often leads to misdiagnosis or missed diagnosis in pediatric patients.

**Methods:**

The clinical data of 11 pediatric patients with KD in the authors' institution were retrospectively analyzed.

**Results:**

There were 11 pediatric patients with KD enrolled in total, including 9 male patients and 2 female patients (sex ratio 4.5:1). The median age at diagnosis stood at 14 years (range 5-18 years), the initial symptoms in all patients included painless subcutaneous masses and focal swelling, the duration of symptoms ranged from 1 month to 10 years, and the average duration was 24.45 months. Six patients had single lesions, and 5 had multiple lesions. The highest proportion of lesion regions were parotid gland (*n* = 5, 31.3%) and retroauricular (*n* = 5, 31.3%), followed by cervical lymph nodes (*n* = 4, 25%), and others (*n* = 2,12.5; elbow *n* = 1; back *n* = 1). The eosinophil absolute count elevated in all patients, ranging from 0.71×10^9^/L to 10.35 ×10^9^/L (normal range 0.02-0.52×10^9^/L). IgE levels were increased in all 7 patients who underwent serum immunoglobulin examination (normal range <100 IU/mL). Three patients received oral corticosteroid treatment while 2 relapsed. Three patients received surgical resection combined with oral corticosteroid treatment, and no patient relapsed. The other 3 patients received surgery and radiotherapy, surgery combined with corticosteroid and cyclosporin and corticosteroid combined with leflunomide respectively, and no patient relapsed.

**Conclusion:**

Base on the study, it is found that Kimura disease is rare and may have the atypical symptoms in pediatric patients, combination therapy is recommended to reduce recurrence, and long-term follow-up should be performed.

## Introduction

Kimura disease (KD), also known as eosinophilic hyperplastic lymphoid granuloma, is a rare benign chronic inflammatory condition, characterized by a painless progressive mass located in the subcutaneous area of the head and neck region, elevated peripheral blood eosinophils, and increased serum immunoglobulin E (IgE) levels (1, 2). It is most often diagnosed in Asian males (20–40 years of age) and has a lower incidence in children (3, 4). At present, there are no recognized diagnostic criteria for KD yet, and histopathological examination is the primary diagnostic method. Moreover, clinical features, laboratory examination, and imaging examination should be considered in the diagnosis. Histopathologically, the main manifestation can be summarized as lymphoid follicular hyperplasia with prominent germinal centers and various degrees of eosinophil infiltration (5). It can also be accompanied by fibrosis, sclerosis, angiogenesis, and eosinophilic abscess (6). KD is rare in clinical practice, especially with low incidence in children, which often brings misdiagnosis or missed diagnosis in pediatric patients. Therefore, it is imperative to conduct related research, and a retrospective analysis is conducted in this study based on the clinical data of 11 pediatric patients with KD. It is hoped that this research will provide more information for the clinical features, diagnosis, and treatment methods in children.

## Materials and methods

In this study, a retrospective analysis was carried out on KD patients admitted to Zhengzhou University's First Affiliated Hospital between January 2010 and October 2022. Inclusion criteria were as follows: (i) patients were confirmed as KD by pathological examination of the masses, presenting as follicular hyperplasia, active germinal centers, eosinophils infiltrate, and so on; auxiliary diagnostic criteria included elevated peripheral eosinophil absolute count and increased serum IgE level; (ii) patients were ≤18 years old. Exclusion criteria involved (i) patients being >18 years old; (ii) not diagnosed at the authors’ institution; (iii) having incomplete clinical data. Clinical data were extracted from electronic medical records, including demographics (age and gender), clinical manifestations, laboratory examinations, imaging and histopathological examinations, treatments, and response to therapy.

## Results

### Clinical features

There were a total of 11 pediatric patients with KD, including 9 male patients and 2 female patients (sex ratio 4.5:1). Their detailed information is shown in Table 1. The median age at diagnosis was 14 years (range 5–18 years). The initial symptoms in all patients included painless subcutaneous masses and focal swelling. The duration of symptoms ranged from 1 month to 10 years, and the average duration is 24.45 months. Six patients had single lesions, and five had multiple lesions. The highest proportion of lesion regions were parotid (*n* = 5, 31.3%) and retroauricular gland (*n* = 5, 31.3%), followed by cervical lymph nodes (*n* = 4, 25%) and others (*n* = 2, 12.5%; elbow *n* = 1; back *n* = 1). The major complications included rash, manifested as red miliary papules scattered on the extremities (*n* = 1, 9.1%) and nephrotic syndrome (*n* = 1, 9.1%). Table 2 displays the summary of the clinical features. To be specific, the eosinophil absolute count elevated in all patients ranging from 0.71 × 10^9^/L to 10.35 × 10^9^/L (normal range 0.02–0.52 × 10^9^/L). IgE levels were increased in all seven patients who underwent serum immunoglobulin examination (normal range <100 IU/mL). None of the aforementioned children had any comorbidity or specific medication history.

**Table 1:** Clinical feature, treatment and result of 11 pediatric patients with KD.

No.	Age (year) / gender/duration (month)	Characteristic of the mass		Blood eosinophilia (×10^9^/L)	Serum IgE (IU/mL)	Treatment	Complication	Recurrence
		Site	Size (cm)					
1	11/M/1	Elbow (L)	4.2 × 1.5	3.47	>2,500	Prednisone + leflunomide	Rash	No
2	14/M/7	Retroauricular (B), cervical lymph node (B)	3.4 × 1.8	4.25	>2,500	Surgery + prednisone + cyclosporin	−	No
3	13/M/36	Parotid gland (L), cervical lymph node (L)	2.2 × 1.1	2.58	−	Surgery + prednisone	−	No
4	14/M/120	Retroauricular (B)	3.0 × 0.9	3.82	>2,500	Prednisone	−	Yes
5	15/M/1	Parotid gland (R)	1.4 × 1.2	1.01	1,687	Surgery + radiotherapy	−	No
6	15/M/12	Retroauricular (R), cervical lymph node (R)	2.6 × 1.8	2.94	−	Prednisone	−	No
7	16/M/24	Parotid gland (R)	6.7 × 3.5	9.3	−	Surgery + prednisone	−	No
8	18/F/10	Parotid gland (R)	3.0 × 2.7	0.71	1,997	Surgery + prednisone	Nephrotic syndrome	No
9	5/F/4	Back (R)	20.6 × 4.4	10.35	4,368	Methylprednisolone	−	Yes
10	6/M/6	Retroauricular (R), parotid gland (R)	3.0 × 2.0	1.84	−	Surgery	−	Yes
11	8/M/48	Retroauricular (B), cervical lymph node (B)	1.5 × 0.8	1.46	2,858	Surgery	−	No

KD, Kimura disease; +, present; −, absent; M, male; F, female; R, right; L, left; B, both; IgE, immunoglobulin E.

**Table 2:** The summary of clinical features.

Characteristics	No. of patients (%)
**Gender**	
Male	9 (81.8%)
Female	2 (18.2%)
**Multiplicity**	
Single	6 (54.5%)
Multiple	5 (45.5%)
**Location**	
Parotid gland	5 (31.3%)
Retroauricular	5 (31.3%)
Cervical lymph nodes	4 (25%)
Others	2 (12.5%)
**Complication**	
Rash	1 (9.1%)
Nephrotic syndrome	1 (9.1%)

### Imaging examination

Ten patients underwent ultrasound examination. The masses located in enlarged lymph nodes and parotid glands mainly appeared as round or oval echo. In addition, thickened cortex and reduced or absent medulla were exhibited in the lymph nodes. The masses in the soft tissue exhibited as hypoechoic or mixed echo, with blurred boundaries. Six patients underwent computed tomography (CT) examination. Most of them showed the enlarged local tissue shadow with uniform density, which were more obvious after the enhanced scan, while only one patient with the mass in the parotid gland showed uneven density and blurred boundary. No significant necrosis or calcification was observed. Magnetic resonance imaging (MRI) was performed on five patients. It showed irregular nodular or clumpy signals, with hypointense or hyperintense on T1-weighted images and hyperintense on T2-weighted images.

### Histopathology examination

**Figure 1:**
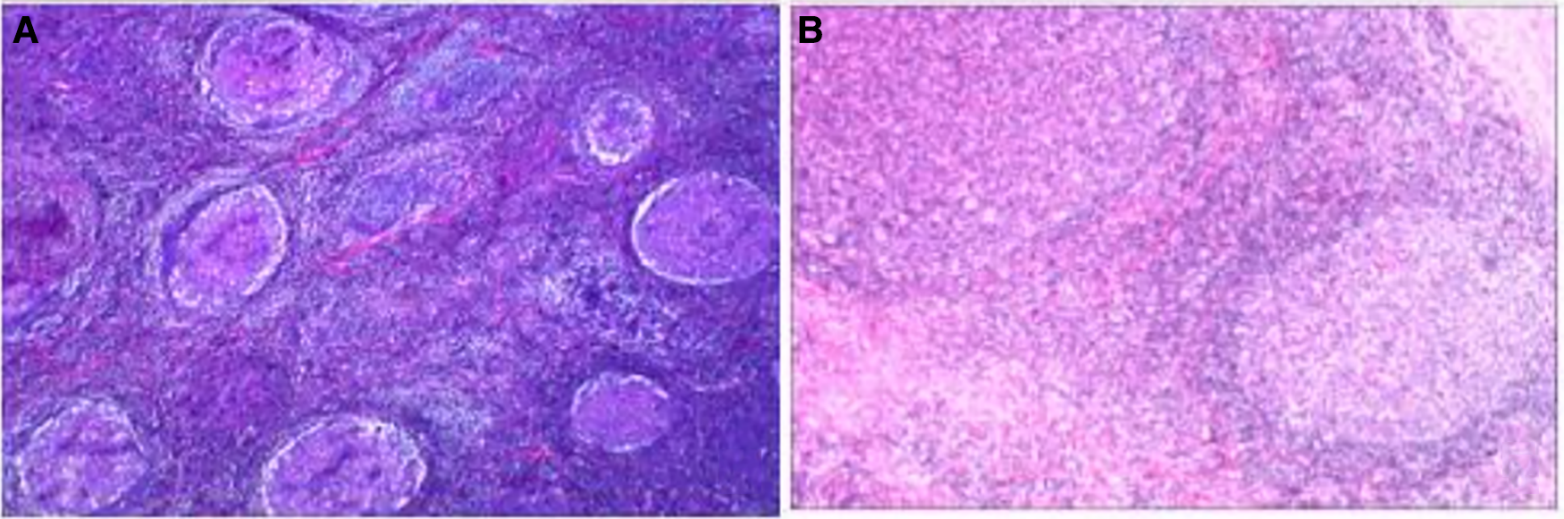
(**A**) Hyperplasia of lymphoid follicular tissue in lymph nodes. (**B**) Reactive germinal center with the obvious well-formed mantle area.

All patients were confirmed by histopathology, whose results were consistent with typical pathological features. Lymphoid follicular hyperplasia was observed in all specimens with extensive eosinophilic infiltration in the follicular space (Figure 1). Reactive germinal centers were observed in eight specimens and eosinophilic microabscesses were observed in five specimens. A crystalline structure, resembling a Charcot–Leyden crystal, was found in one case. Immunohistochemical staining was performed in seven cases, with positive staining for CD3, CD20, Ki-67, Bcl-2, and Bcl-6, and negative staining for S-100 and CD1a. Furthermore, five patients received bone marrow aspiration, which showed active bone marrow proliferation, granulocytic hyperplasia with normal morphology, and eosinophilia. The patient with nephrotic syndrome received ultrasound-guided renal biopsy, which showed minimal change disease.

### Treatment and recurrence

Among the 11 cases, 2 underwent surgical resection and 1 relapsed whose recurrence location (left retroauricular) was different from the initial location (right retroauricular and parotid gland). Three patients received oral corticosteroid treatment (prednisone 0.5–0.7 mg/kg day and methylprednisolone 0.5 mg/kg day, and the dose was gradually reduced as the masses shrank) while two of them relapsed. Three cases received surgical resection combined with oral corticosteroid (prednisone) treatment, and no case relapsed. The other three patients received surgery and radiotherapy, surgery combined with corticosteroid (prednisone) and cyclosporin, and corticosteroid (prednisone) combined with leflunomide (0.5 mg/kg day), respectively, and no case relapsed. The information on treatment and recurrence is demonstrated in Tables 1 and 3, respectively. During the follow-up period (ranging from 3 to 91 months), 3 of the 11 patients relapsed in total, with a mean recurrence time of 16.7 months whose peripheral blood eosinophil and IgE levels increased again to varying degrees with recurrence. The patients with recurrence were treated with methotrexate, cyclosporine, and surgical resection, respectively, and none of them have recurred until now.

**Table 3:** Treatment modalities and recurrence rate.

Treatment	No. of patients	No. of recurrences (%)
Surgery	2	1 (50.0%)
Corticosteroid	3	2 (66.7%)
Surgery + corticosteroid	3	0
Surgery + radiotherapy	1	0
Surgery + corticosteroid + cyclosporin	1	0
Corticosteroid + leflunomide	1	0

## Discussion

Kimura disease was originally described by Chinese surgeons Kim and Szeto and was named after “eosinophilic proliferative lymphogranuloma” in a Chinese journal in 1937. Later, in 1948, Japanese scholars Kimura et al. reported the related cases with the title “*On the Unusual Granulation Combined with Hyperplastic Changes of Lymphatic Tissue*” and described its definitive histopathological features, which later became widely known as Kimura disease (5). KD is more common in young Asian males with a sex ratio of 3.5–9:1 (7). In a previous report reviewing all cases from 1998 to 2018, it was revealed that the sex ratio was 17:1 for <20 years, and the proportion of affected female patients increased with age (8). In another study of pediatric patients, the sex ratio was 28:1, and the median age at diagnosis reached 12 years (ranging from 2.7 to 18 years) (4). In this study, the median age at diagnosis was 14 years, ranging from 5 to 18 years, similar to previous studies. However, the male-to-female ratio was 4.5:1, which might be attributed to the small sample size. This condition is characterized by the concealed onset and a long course. The most frequently affected areas are the head and neck region, especially the parotid glands, salivary glands, and lymph nodes (9). Masses in other areas, such as the groin (10), breast (11), arm (12), thigh (13), and orbit (14), have also been reported. This study was consistent with previous studies that most lesions (14/16) were located in the head and neck region, but there were still two cases located in the back and elbow, respectively. To the best of our knowledge, this research involves the first case of KD starting with a back mass reported in the English literature, especially in a female pediatric patient.

At present, the etiology and pathogenesis of Kimura disease remain unclear. Various theories have been postulated to explain, but none have been confirmed. Currently, the widely accepted possible hypothesis is that infection and parasitic triggers such as arthropod bites, Epstein–Barr virus, human herpesvirus 8, human polyomavirus 6, and *Candida albicans* alter T cell immunoregulation or induce IgE-mediated type 1 hypersensitivity (6, 15). Elevated eosinophils in peripheral blood and bone marrow and increased serum level of IgE in KD patients suggest that T helper 1 and 2 (Th1 and Th2) cells and T regulatory cell may be involved in the pathogenic process of KD (16). Ohta et al. detected T cell subsets by flow cytometry and found that Th2 cells in the KD patient group were significantly higher than those in the control group (17). Katagiri et al. found that TNF-α, IL-4, IL-5, and IL-13 mRNA levels were significantly elevated in patients with Kimura disease before treatment and decreased after surgery or radiotherapy, in which supported Th2 cytokines play a role in the development of KD (18). IL-4, IL-5, and IL-13 can promote the increase and aggregation of eosinophils and induce B cells to preferentially express IgE (16, 17). In this study, eosinophils were elevated in all patients (11/11), and serum IgE was significantly elevated in all 7 patients with IgE detection.

Pruritus, rash, eczema, and urticaria are common concomitant symptoms of this disease, which may be the result of eosinophils infiltration and cytokine release (19). In this study, one patient presented with rash that resolved spontaneously after treatment for KD. Nephrotic syndrome is also associated with KD, and its pathogenesis may be related to IgE deposition in glomeruli, eosinophilic infiltration, and changes in basement membrane permeability by cytokines (20). The review by Yamada et al. revealed that nephrotic syndrome accounted for 7.4% (13/175) of patients with KD (21, 22). In the study of KD pediatric patients by Xu et al. the incidence of nephrotic syndrome was higher, at 27.6% (4). Renal involvement usually occurs after subcutaneous masses or lymphadenopathy, but it has also been mentioned to occur first or simultaneously (7, 23). One patient was found to have minimal change nephrotic syndrome, almost co-occurring with the subcutaneous mass. The proportion of kidney damage in children with KD may be higher than that in adults. Therefore, routine examination of urine and renal function should be performed in time after diagnosis, so as to improve the prognosis of nephropathy by early treatment.

Imaging examinations are not specific but are helpful to determine the feature, location, and boundary of the masses. In a previous review of ultrasound reports from 58 patients with KD, the mass presented as ill-defined, solid, and heterogeneous echogenic lesions, and blood flow signals could be found partially. Enlarged hilum of lymph node was seen in all involved lymph nodes (24). Gopinathan and Tan classified KD into three types according to the CT images: type 1, the nodular mass with well-defined borders and uniform enhancement; type 2, the patchy lesion of varying degrees of enhancement with an ill-defined boundary; type 3, with the above two lesions at the same time (25). In this paper, among the six patients who underwent CT examination, only one patient presented with type 2, while the rest presented with type 1. A previous systematic review has reported that the lesions showed equal to high intensity on T1-weighted images and heterogeneously high signal on T2-weighted images on MRI, which was consistent with our findings (24). The slight differences in image features could be ascribed to the different degrees of vascular proliferation and fibrosis in the primary lesion (26). In addition, the enhancement degree of MRI images after enhancement was generally higher than that of CT images, indicating that MRI has a higher sensitivity to the changes after enhancement (27).

Based on the above clinical manifestations, laboratory tests, and imaging tests, KD can be initially considered, while pathological examination is the gold standard for diagnosis. The most typical pathological manifestations include inflammatory cell proliferation and infiltration, lymphoid follicular hyperplasia with will-formed mantle zones and reactive germinal centers, numerous eosinophils infiltration in the interfollicular space, proliferation of postcapillary venules, and the formation of eosinophilic microabscesses. The pathological examination of the patients with a back mass reported some crystalline structures, like the Charcot–Leyden crystal, accompanied by lots of microabscesses. It was considered that the mass was probably too large and a large number of eosinophils gathered and were necrotic. In this report, immunohistochemical staining was examined to be positive for CD3, CD20, Ki-67, Bcl-2, and Bcl-6, and negative for S-100 and CD1a, which was similar to previous reports (28, 29).

Angiolymphoid hyperplasia with eosinophilia (ALHE), Langerhans cell histiocytosis, and lymphoma need to be differentiated from KD. Because of the similar histopathology and clinical features, KD and ALHE were once deemed as the same disease. Compared with KD, ALHE usually occurs in middle age female (20–50 years) without a significant racial difference, and the eosinophils and IgE in peripheral blood are not significantly increased, with no association with kidney damage. The main histopathology can be depicted as the vascular proliferation of atypical endothelial cells (1, 30). Langerhans cell histiocytosis is a rare benign disease that accumulates in bones, skin, lungs, and lymph nodes, which is more common in children. Pathological examination revealed that the lesions were characterized by “coffee bean” like Langerhans cell proliferation, which was not found in KD, accompanied by scattered eosinophils infiltration, and immunohistochemistry showed that S100 and CD1a were tested to be positive by immunohistochemistry (31, 32). Lymphoma, as a large group of malignancies that are common in children, shares commonality with KD and, therefore, should be primarily differentially diagnosed. With chronic progressive painless lymph node enlargement as the main clinical manifestation, Hodgkin's lymphoma mostly starts with masses located in the neck, similar to KD. It is generally identified by pathology: tumor cells, such as Hodgkin and Reed–Sternberg (HRS) cells, are scattered in a large number of immune cells. Other types of lymphoma, such as B-lymphoblastic lymphoma, can all be identified by abnormalities in cell morphology and immunophenotype. It should be added that IgG4-related diseases, Castleman's disease, parasitic lymphadenitis, and Churg–Strauss syndrome can be easily mixed up with KD.

There are several treatment options for KD, including surgical resection, corticosteroids, radiotherapy, immunosuppressive agents, and biologics, with varying degrees of efficacy. Surgical resection is usually the preferred treatment, but it is difficult to completely remove the tumor due to the large size, unclear boundary, and its recurrence rate is high (30.5%–100%) (33, 34). Oral corticosteroid, immunosuppressive agents, and other drug treatments usually tend to be effective on KD, but patients are more likely to relapse after drug withdrawal (35). In this study, of all three recurrent patients, one was treated with surgical resection alone, and two received oral corticosteroid therapy alone. Local radiation therapy is impressive in treating recurrent or persistent lesions with a high local control rate and is also recommended in patients with positive surgical margins and repeated postoperative recurrences (36, 37). Ye et al. argued that there was a lower local recurrence rate in patients with KD treated by surgery combined with radiotherapy (38). However, it is not recommended for pediatric patients given its growth retardation and carcinogenic side effects (39). Some current studies have pointed out that surgical resection or oral corticosteroid therapy combined with other adjuvant treatments, such as immunosuppressive therapy (azathioprine, cyclophosphamide, cyclosporine, mycophenolate mofetil), achieved better treatment results and lower recurrence rates (40). A systematic review revealed that recurrence rates registered 30.5%, 45%, and 60% for surgical resection, drug therapy, and radiation therapy alone, respectively, while the lowest recurrence rate stood at 26.94% for combined therapy (34). In this study, six patients received combination therapy, and no recurrence has been reported until now, which is in line with the previous results. Several biological agents, such as benralizumab (41), an anti-IL-5 receptor antibody; mepolizumab, an anti-IL5 antibody (42); and dupilumab, a Th2 pathway-targeted agent (13), have been successfully used in the treatment of KD on a small scale, but the efficacy in pediatric patients still needs further validation.

As a benign disease, KD has a good prognosis, but the recurrence rate, which is independent of age, is high (24%–62%) (8, 43). In a recent meta-analysis, it was noted that KD patients with a maximum tumor diameter ≥3 cm, a disease duration ≥5 years, a peripheral blood eosinophil count ≥20%, or a serum IgE level ≥10,000 IU/mL were more likely to recur after surgical resection (34). Furthermore, single surgical resection or corticosteroid therapy, ill-defined margins, and the multiple regions involved were associated with disease recurrence (4, 44). The mean time of recurrence after the initial treatment can reach 3.7 years, so KD patients should be followed up for a long time, and combination therapy should be given priority after recurrence (43).

This study demonstrated several deficiencies and strengths. In this paper, we elaborated on the clinical features, examination, treatment, and prognosis of 11 pediatric patients, who are less common in KD. However, this was a single-center retrospective study with a relatively small sample size. The treatment regimens and recurrence rate were hence limited. More prospective, multicenter, large-scale studies are needed.

## Conclusion

Since Kimura disease is rare and may present atypical symptoms in pediatric patients, attention should be paid to the differential diagnosis of the prevalent malignant diseases in children, and the diagnosis should be supported by pathological tests. In terms of treatment, combination therapy is recommended to reduce recurrence, long-term follow-up needs to be improved, and complications such as nephrotic syndrome deserve more attention.

## Data Availability

The original contributions presented in the study are included in the article/Supplementary Material, further inquiries can be directed to the corresponding author.
